# North American Genetic Counselors' Perceptions of Identifiability From Demographic Questions on Surveys

**DOI:** 10.1002/jgc4.70292

**Published:** 2026-09-15

**Authors:** Aarti Ramdaney, Jennifer Czerwinski, Myla Ashfaq

**Affiliations:** ^1^ Department of Obstetrics Gynecology and Reproductive Sciences, McGovern Medical School at the University of Texas Health Science Center at Houston Houston Texas USA; ^2^ Division of Medical Genetics, Department of Pediatrics McGovern Medical School at UTHealth Houston Houston Texas USA

**Keywords:** demographics, genetic counselors, identifiability, professional issues, surveys, underrepresented minority groups, underrepresented populations

## Abstract

Demographic data are routinely collected in surveys to assess participant diversity, analyze subgroup trends, and promote ethical, representative research. However, in small professional fields like genetic counseling, the combination of multiple demographic variables can raise concerns about identifiability. This study aimed to examine whether the design of demographic questions affects the willingness of genetic counselors (GCs) in the United States and Canada to participate in surveys and to respond accurately, particularly those who identify as part of an underrepresented minority (URM) group. A total of 153 GCs completed a 13‐item online survey between August and September 2023. The survey included questions assessing whether participants identified as part of an URM within the profession, their level of participation in research and professional surveys, and concerns with demographic questions and the potential to be identified. Quantitative data were analyzed using descriptive statistics in Microsoft Excel, and qualitative free‐text responses were examined using inductive thematic analysis to identify common concerns and recommendations. Respondents who identified as part of an URM were more likely to feel that they could be identified based on their responses to demographic responses compared to their non‐URM peers. Although all participants reported active participation in research and professional surveys, several indicated they skipped certain demographic questions or modified their responses to increase anonymity. In open‐ended responses, participants emphasized the importance of limiting unnecessary demographic questions and using flexible formats to minimize identifiability. Our study highlights the need for greater reflection on the design of demographic questions as they can affect how willing GCs are to take part in surveys, especially those from URM backgrounds, and overall data quality and representation. We encourage researchers to be transparent about why demographic information is collected, offer flexible and inclusive response options, and assess ways to protect anonymity as it may help build trust and encourage fuller participation in research.

## Abstract (highlights)

**What Is Known About This Topic:**

Research and professional surveys collect demographic information from the study participants for many reasons. For individuals who identify as part of an underrepresented minority group, this may lead to concerns with identifiability. The perception of identifiability has been shown to reduce willingness to participate or limit full disclosure.

**What this paper adds to the topic:**

Genetic counselors who identify as part of underrepresented minority groups are more likely to feel they could be identified based on their demographic responses, leading some to skip questions, discontinue surveys, or misreport their demographics. Researchers should reflect on the need and/or relevancy of specific demographic questions in the context of the study topic, and provide clarity to how data will be used.

## 1 Introduction

The field of genetic counseling has long grappled with issues of diversity and representation among its members. Year over year, the National Society of Genetic Counselors (NSGC) Professional Status Survey (PSS) describes the profession as predominantly composed of individuals who identify as female, White, and able‐bodied (Channaoui et al. 2020; Mittman and Downs 2008; National Society of Genetic Counselors 2024). In response to these disparities, the NSGC launched its Justice, Equity, Diversity, and Inclusion (J.E.D.I.) initiative in 2022, aimed at fostering a more inclusive and supportive environment of the organization and promoting the development of a more diverse workforce of genetic counselors (National Society of Genetic Counselors, n.d.). Still, while efforts to promote diversity are ongoing, the lived experiences of those identifying as an underrepresented minority (URM), including racial and ethnic minorities, gender‐diverse individuals, and those with disabilities, remain underexplored in the context of research and professional survey practices.

Research and professional surveys typically collect demographic information from the study participants for several reasons. One common purpose is to analyze whether a person's identity influences specific behaviors or whether external factors shape aspects of identity (Hughes et al. 2016). Demographic information also helps researchers describe the study sample, assess the generalizability of findings, ensure that diverse populations are adequately represented in data analyses, and support subgroup analyses to potentially uncover underlying disparities (National Institutes of Health, n.d.; Polit and Beck 2010). Accurate demographic information is therefore critical to ensuring that research findings are interpretable, generalizable, and capable of identifying meaningful differences across diverse populations.

Although there has been growing attention to improving the inclusivity and accuracy of demographic data collection in social science and health research (Moreira et al. 2023), limited attention has been given to how these issues affect the genetic counseling community. To date, no studies have specifically examined how genetic counselors (GCs) from underrepresented groups experience or respond to demographic questions in research. According to the 2024 NSGC PSS, there are approximately 6900 certified GCs in the United States as of April 2024. With less than 700 students matriculating into genetic counseling graduate programs in 2026 (National Matching Services Inc. n.d.), the profession remains relatively small and lacks substantial diversity. As a result, concerns about identifiability may emerge when surveys request demographic details such as age, race, year of graduation, or training program.

These considerations raise important questions about the extent to which GCs are willing to provide demographic information in research studies. Do GCs respond to all demographic questions comprehensively and accurately, or do they intentionally skip questions, provide inaccurate information, or opt out of the survey altogether due to concerns about being identified? Given the high volume of surveys distributed to GCs and the associated risks of respondent fatigue and nonresponse bias (Ben‐Nun 2008; Myers and Atzinger 2025), such behaviors may further exacerbate data quality issues by increasing rates of item nonresponse, misreporting, or total survey nonresponse, ultimately introducing bias and reducing the representativeness of the data. This study explores whether the design of demographic questions affects the willingness of GCs in the United States and Canana from underrepresented groups to participate in surveys and to respond accurately. By centering the perspectives of GCs from underrepresented backgrounds, this work seeks to guide the development of more equitable and methodologically sound surveys in genetic counseling, aligning data collection practices with the broader values of inclusion and representation.

## 2 Methods

This cross‐sectional study was approved by the Institutional Review Board at the University of Texas Health Science Center at Houston (HSC‐MS‐23‐0599).

### 2.1 Participants and Recruitment

All Full, New Genetic Counselor, and Emeritus NSGC members, American Board of Genetic Counseling (ABGC) diplomates, and Canadian Association of Genetic Counselors (CAGC) members 18 years and older were eligible to participate. Individuals did not have to identify as part of an URM group to qualify for participation. An invitation to the online survey was distributed through the NSGC, ABGC, and Texas Society of Genetic Counselors (TSGC) research blasts. An invitation was also distributed through the Minority Genetics Professional Network (MGPN). Data collection spanned from August to September 2023.

### 2.2 Instrumentation

A secure online data collection and survey tool, Redcap, was used to create the survey and store responses (Harris et al. 2019). Informed consent was obtained from all eligible participants by selecting “agree” after reading the study information on the first page of the online survey. The survey contained 13 questions, primarily multiple‐choice style questions. Participants were asked to confirm if they were an NSGC member, ABGC diplomate, and/or CAGC member, as well as note if they identified as part of an URM group within the genetic counseling profession. A definition for groups that may be considered an underrepresented minority was intentionally not provided to allow participants to self‐assess and not be limited by the perception of others. All participants were asked to select what aspect(s) of their identity felt underrepresented within genetic counseling, including their age, age at professional entry, if they were a member of the disability community, gender identity, political affiliation, race or ethnicity, religious and/or spiritual beliefs, sexuality, and socioeconomic status. Participants also could select “other” and submit their perceived aspect(s) via free text or indicate that they did not feel that any aspects of their identity felt underrepresented. Because perceptions of underrepresentation may differ from predefined demographic classifications, discrepancies between reported URM status and selected underrepresented identity characteristics were retained as reported and were not corrected or reclassified. Each survey item was analyzed independently based on participants' self‐reported responses.

Survey questions assessed participants' frequency of participation in research studies and/or professional status surveys, with response options including “Yes, often,” “Yes, sometimes,” “I used to, but no longer participate in any surveys,” and “No.” Participants were also asked if their responses to demographic questions ever made them feel identifiable and, if so, to select which factors made them feel identifiable. Potential factors participants could choose from included none, age, age at professional entry, being a member of the disability community, gender identity, graduate program, job title, location or region of residence, political affiliation, practice location, race or ethnicity, religious and/or spiritual beliefs, sexuality, specialty, year of graduate school graduation, and other. Using a 10‐point Likert scale where 1 = not at all concerned and 10 = very concerned, respondents rated their level of concern about being personally identifiable based on survey demographic responses as well as how concerned they would be if others were identifiable. An additional question explored participants' motivations in completing surveys, including if they completed surveys if they felt identifiable and if they changed demographic responses or skipped certain questions to reduce the chance of being identified. Participants were also asked what demographic questions they felt willing to answer routinely. Finally, two open‐ended questions invited participants to reflect on what circumstances, if any, would change their willingness to answer demographic questions and to share their perspective on how they felt demographic information should be collected and/or utilized in research focused on GCs.

The survey was developed by the authors (AR, JC, MA). Each author provided revisions and input prior to the survey being pre‐tested by three current clinical GCs, two of whom were also members of MGPN. Based on this feedback, revisions were made to clarify the concept of identifiability in the context of the research question and add other demographic factors that may be viewed as underrepresented within the professions.

### 2.3 Data Analysis

Survey responses were extracted from RedCap and analyzed using both quantitative and qualitative methods. Descriptive statistics were used to summarize participant characteristics and survey responses. Categorical variables were summarized using frequencies and percentages. For survey items allowing multiple responses, each response option was analyzed as a separate binary variable, with percentages calculated using the total number of respondents. Associations between perceived URM status (yes, no, unsure) and categorical survey responses were evaluated using Pearson's chi‐square tests, with Fisher's exact tests used when expected cell counts were small; Fisher's exact *p*‐values are reported. Analyses were conducted using available‐case analysis, with participants excluded only from analyses for survey items with missing responses. Statistical tests were performed using Stata v17. A *p* value < 0.05 was considered statistically significant.

Open‐ended responses were coded for themes using inductive thematic analysis in Excel (Braun and Clarke 2006). Authors AR and MA independently reviewed all open‐ended responses and identified recurring themes. Discrepancies in identified themes were resolved through discussion until a consensus was reached.

## 3 Results

A total of 153 individuals completed the survey, and the majority identified as full NSGC members (128/153, 82.4%). Because the survey was disseminated through multiple professional organizations with overlapping memberships, the number of unique individuals who received the survey invitation could not be determined. Consequently, an accurate response rate could not be calculated. Using the published number of members in NSGC in September 2023 as a proxy denominator, we would estimate a response rate of 3.1% (153/4951) (Babu 2023). All respondents in our cohort reported participating in research surveys and/or professional surveys, with a slight majority (79/153, 51.6%) indicating that they often participate and the remaining (74/153, 48.4%) reporting that they sometimes participate.

Almost 40% (61/153, 39.9%) respondents self‐identified as belonging to an URM group within the genetic counseling profession, while 53.6% (82/153) did not, and 6.5% (10/153) were unsure. The most commonly selected aspects that were perceived to be unrepresented were sexuality (32/153, 20.9%), race/ethnicity (31/153, 20.3%), religious/spiritual beliefs (23/153, 15.0%), and gender identity (16/153, 10.5%). Respondents were able to choose multiple aspects, and 30.7% (47/153) noted having more than one aspect underrepresented in the profession. Additional demographic characteristics of the study cohort are available in Table 1.

**TABLE 1:** Participant demographics.

Characteristics	*n* = 153	%
**Professional association**		
New NSGC member	21	13.7
Full NSGC member	126	82.4
Emeritus NSGC member	0	0.0
ABGC member	58	37.9
CAGC member	3	2.0
**Identify as underrepresented minority**		
Yes	61	39.9
No	82	53.6
Unsure	10	6.5
**Perceived underrepresented aspects**		
Age	8	5.2
Age at professional entry	12	7.8
Disability community member	17	11.1
Gender identity	16	10.5
Political affiliation	10	6.5
Race/ethnicity	31	20.3
Religious/spiritual beliefs	23	15.0
Sexuality	32	20.9
Socioeconomic status	11	7.2
Other	5	3.3
None	61	39.9
**Number of aspects selected**		
0	61	39.9
1	45	29.4
2	29	19.0
3	12	7.8
4	4	2.6
5	2	1.3
**Survey participation**		
Yes, often	79	51.6
Yes, sometimes	74	48.4

When asked whether they had ever felt their responses to demographic questions in genetic counseling surveys could reveal their identity, 102/150 (68.0%) noted that they felt they could be identified while 48/150 (32.0%) did not feel that they could be identified; three participants did not respond to this question. Respondents who identified as part of an URM were more likely to report feeling identifiable based on their demographic responses compared with respondents who did not identify as a part of an URM or were unsure (78.7% [48/61] vs. 58.5% [48/82] and 60.0% [6/10], respectively; Fisher's exact test, *p* = 0.002). The most frequently selected demographics that made participants feel identifiable were year of graduation (*n* = 71), location/region of residence (*n* = 61), graduate program (*n* = 63), and practice location (*n* = 57). See Table 2. Those participants who were part of the URM population based on being a member of the disability community (*p* < 0.001), their gender identity (*p* < 0.001), race/ethnicity (< 0.001), religious/spiritual beliefs (*p* = 0.042), and sexuality (p = 0.002) felt significantly more identifiable as an URM compared to those that did not or were unsure.

**TABLE 2:** Participants' perceptions of identifiability.

	Identify as URM		Do not identify as URM		Unsure	%	*p*
	*n* = 61	%	*n* = 82	%	*n* = 10		
**Perceived identifiability based on responses**							0.002
No	13	21.3	33	40.2	2	20.0	
Yes	48	78.7	48	58.5	6	60.0	
Unknown	0	0.0	1	1.2	2	20.0	
**Identifiable demographics**							
Age	13	21.3	14	17.1	2	20.0	0.716
Age at professional entry	8	13.1	9	11.0	2	20.0	0.632
Disability community member	9	14.8	0	0.0	0	0.0	< 0.001
Gender identity	12	19.7	1	1.2	2	20.0	< 0.001
Graduate program	30	49.2	28	34.1	5	50.0	0.935
Job title	7	11.5	17	20.7	2	20.0	0.313
Location/region of residence	27	44.3	29	35.4	5	50.0	0.446
Political affiliation	1	1.6	0	0.0	0	0.0	0.464
Practice location	22	36.1	31	37.8	4	40.0	0.965
Race/ethnicity	22	36.1	1	1.2	3	30.0	< 0.001
Religious/spiritual beliefs	6	9.8	1	1.2	1	10.0	0.042
Sexuality	10	16.4	1	1.2	1	10.0	0.002
Specialty	21	34.4	20	24.4	3	30.0	0.414
Year of graduation	26	42.6	41	50.0	4	40.0	0.663
Other	6	9.8	3	3.7	9	90.0	0.249
None	9	14.8	25	30.5	10	100.0	0.071

Participants who self‐identified as an URM reported greater concern about being personally identifiable based on demographic responses (mean = 4.4, SD = 2.7) compared to those who did not identify as an URM (mean = 2.8, SD = 2.5) and those who were unsure (mean = 5.4, SD = 2.1). In contrast, concern regarding the identifiability of others was similar across groups (URM: mean = 6.8, SD = 2.1; non‐URM: mean = 7.0, SD = 2.1; unsure: mean = 6.8, SD = 2.1).

Respondents largely felt willing and/or comfortable answering most demographic questions on surveys (Table 3). Overall, willingness in answering demographics was similar for those who identified as part of an URM group compared to those who did not or were unsure. The only significant difference observed was for sexuality‐related demographic questions, for which respondents identifying as URM reported lower comfort answering compared with non‐URM respondents (60.7% vs. 75.6%; Fisher's exact test, *p* = 0.024). To determine whether this association was driven by specific underrepresented identities, additional subgroup analyses were performed examining race/ethnicity, sexuality, and religious/spiritual beliefs. Respondents identifying as an URM due to their sexuality were significantly less willing to answer sexuality questions (*p* = 0.0003), and respondents identifying as an URM due to their race/ethnicity were less willing to answer race/ethnicity questions (*p* = 0.042). There was no significant difference in willingness to answer demographic questions relating to religion/spiritual beliefs for those who identified as an URM due to religious/spiritual beliefs compared to those who did not (*p* = 0.70). Motivations for survey completion when feeling possibly identifiable were similar across all participants, with most selecting interest in the survey topic (115/153, 75.2%), the survey being part of a student project (95/153, 62.1%), and/or financial incentives (48/153, 31.4%). Table 4 provides further details on participant survey completion.

**TABLE 3:** Willingness in answering demographic questions.

	Identify as URM		Do not identify as URM		Unsure	%	*p*
	*n* = 61	%	*n* = 82	%	*n* = 10		
**Willing to answer**							
Age	56	91.8	73	89.0	7	70.0	0.133
Age at professional entry	48	78.7	59	72.0	5	50.0	0.154
Disability community member	33	54.1	45	54.9	4	40.0	0.732
Gender identity	48	78.7	73	89.0	9	90.0	0.198
Graduate program	36	59.0	53	64.6	4	40.0	0.301
Job title	49	80.3	64	78.0	8	80.0	0.949
Location/region	42	68.9	58	70.7	7	70.0	0.960
Political affiliation	30	49.2	47	57.3	4	40.0	0.434
Practice location	32	52.5	48	58.5	6	60.0	0.756
Race/ethnicity	49	80.3	74	90.2	74	740.0	0.326
Religious/spiritual beliefs	41	67.2	59	72.0	5	50.0	0.352
Sexuality	37	60.7	62	75.6	4	40.0	0.024
Specialty	51	83.6	72	87.8	8	80.0	0.576
Graduation year	45	73.8	59	72.0	7	70.0	0.960
Other	1	1.6	1	1.2	0	0.0	1.000
None	1	1.6	1	1.2	0	0.0	1.000

**TABLE 4:** Participant survey completion.

	Identify as URM		Do not identify as URM		Unsure	%	*p*
	*n* = 61	%	*n* = 82	%	*n* = 10		
**Survey completion motivation**							
Interest in topic	51	83.6	57	69.5	7	70.0	0.134
Survey is part of student project	38	62.3	51	62.2	6	60.0	1.000
Interest in topic	51	83.6	57	69.5	7	70.0	0.134
Survey is part of professional org	15	24.6	17	20.7	4	40.0	0.346
Rewards or financial incentives	22	36.1	22	26.8	4	40.0	0.431
Do not complete/exit survey	8	13.1	11	13.4	2	20.0	0.751
Complete survey, but change responses	13	21.3	15	18.3	1	10.0	0.759
Other	6	9.8	8	9.8	0	0.0	0.823
**Factors influencing exit of survey**							
Number of questions	2	3.3	4	4.9	0	0.0	1.000
Type of questions	8	13.1	8	9.8	1	10.0	0.840
Topic of study	3	4.9	3	3.7	1	10.0	0.436
Flow of questions	1	1.6	4	4.9	1	10.0	0.219
Other	1	1.6	3	3.7	0	0.0	0.723
**Participant changing response**							
Skip question if not required	10	16.4	15	18.3	1	10.0	0.944
Choose another option randomly	1	1.6	1	1.2	0	0.0	1.000
Choose what “majority” may select	4	6.6	0	0.0	0	0.0	0.062
Other	2	3.3	1	1.2	0	0.0	0.654

Twenty‐one (13.7%) participants noted that they did not complete or they exited a survey if they felt identifiable, and the most common factors influencing exit of a survey were the type of questions and topic of study. Twenty‐nine (19.0%) participants selected that they completed the survey but would change responses or skip certain questions to reduce the chance of being identified. Among these participants, the most frequent strategy was to skip non‐mandatory questions (*n* = 26). Others noted that they would choose an option that they felt the “majority” would select (*n* = 4) or choose a different option to the question randomly (*n* = 2). There was no statistical difference found with responses, though individuals who identified as an URM were somewhat more likely to select that they would change their response to what the “majority” may select compared to those who did not identify as an URM or were unsure (*p* = 0.062).

Open‐ended responses regarding circumstances that may influence what demographic questions participants routinely answered revealed several themes (Table 5). The most prominent theme was the nature of the specific question being asked, followed by the sample size of the study and the survey topic. Fourteen participants reported being unconcerned about being personally identified, though some acknowledged that this lack of concern may reflect their self‐perceived privilege. Other common themes included feeling more concerned when a combination of demographic questions was asked and if there could be situations with the potential for professional harm if identified.

**TABLE 5:** Themes identified from open‐ended responses on factors/circumstances influencing willingness to answer demographic questions.

Themes	Illustrative quotation(s)	% (*n*) total responses = 79
Concern with specific demographic questions	“My graduate program is fairly new and lists all past students and where they currently work. If this information were less publicly available, I would be more willing to answer questions about where I went to school.”	26.6% (21)
Sample/population size limitations	“I think if there were more trans/nonbinary/gender‐diverse genetic counselors I would be less worried that I could be identified.”	21.5% (17)
Study topic	“Depending on the scope of the project and any views I have that be out of the ‘norm’ that would change my want to be identifiable on the survey”	20.3% (16)
Unconcerned about potential to be identified	“Nothing–I am generally pretty open but that might speak more to my privileges afforded to me by being a heteronormative, white, cis male.”	17.8% (14)
Concern with combination of demographic questions	“It is the combination that concerns me. If it was just state/region or just year of graduate program graduation, it wouldn't be a problem, but when you start stacking things it becomes apparent that anyone who is from a smaller % demographic it wouldn't take a rocket scientist to figure out who I am. And I am a person from a place of relative privilege so it has definitely given me pause for people who are URM by race/ethnicity, sexuality, etc. and how they might feel.”	11.4% (9)
Potential for professional harm	“In general, GC surveys have appropriate scrutiny and checks by peer‐reviewers that I feel comfortable sharing my demographics. In rare instances, direct quotes in a survey, as opposed to an aggregate data analysis, might have implications of identifying me that can result in long‐term harm (e.g., loss of employment, judgment from other GCs/HCPs, etc.).”	10.1% (8)

Additionally, respondents had varying thoughts of how demographic information should be collected and/or utilized in research and surveys focused on genetic counselors (Table 6). The most common theme was that demographic questions should be included only if they are necessary for analysis, followed by suggestions to use broader grouping or ranges when collecting demographic data.

**TABLE 6:** Considerations on the collection and use of demographic data in genetic counseling research.

Themes	Illustrative quotation(s)	% (*n*) total responses = 77
Only included if necessary for analysis	“Not all of them [demographic questions] all of the time. Really thinking about WHY you need (or do not need) to know something.” “I think it's best to stick to the minimum amount of demographic information necessary to understand your participants and how representative of a population they are. There's been times where I've felt surveys have asked about certain demographic info just for the sake of asking it, which rubs me the wrong way and makes me not want to complete a survey due to a perceived loss of anonymity—especially as someone that belongs to multiple minority populations and works in a smaller specialty.”	46.8% (36)
Use of grouping/ranges to minimize potential identifiability	“More grouping would make less likely to be identifiable.” “A certain sample size under which results should not be presented or should be grouped into ‘other.’”	16.9% (13)
Collection of specific demographic questions	“As I get older, I think age is a big identifier considering if anyone in my area looked, they would know it was me.”	13.0% (10)
Questions that ensure representation	“I think having demographic information is incredibly important to provide context for the overall findings, like if the majority of participants fall into the majority of the demographic makeup of the GC field at the time, that should prompt further questions/focused attention into how individuals from minority groups in the study responded as well.”	13% (10)

## 4 Discussion

Our findings suggest that perceived identifiability is an important consideration in how GCs respond to demographic questions and may influence willingness to disclose demographic questions, particularly among individuals from URM backgrounds. Although collection of demographic data can be essential for understanding workforce diversity and evaluating efforts to improve equity and inclusion, our results indicate that GCs may weigh the benefits of providing this information against concerns of potential identification, particularly within the context of a relatively small professional community and even in the absence of direct identifiers.

Though Institutional Review Boards (IRBs) play an important role in ensuring that appropriate safeguards for privacy, confidentiality, and informed consent are in place, prior studies have shown that even ‘quasi‐identifiers’ or variables that are not direct identifiers can result in re‐identification of individuals when linked to public membership lists or other data sources (Rocher et al. 2019). Within genetic counseling, a profession with a relatively small and well‐defined membership, the collection of multiple demographic factors, such as year of graduation, training institution, and race/ethnicity, may increase perceived identifiability. In our study, 68% of respondents reported that they felt they could be identified through their responses to demographic questions when completing genetic counseling‐related surveys. Though our study assessed perceived rather than actual identifiability risk, perceptions of identifiability may still influence participant behavior regardless of the actual likelihood of identification. Notably, participants in our study most frequently identified professional characteristics, such as training program, graduation year, and geographic location, rather than personal identity characteristics alone, as contributing to this concern. We hypothesize that these professional characteristics may function as quasi‐identifiers that can be perceived as identifying when considered in combination with one another. Moreover, individuals belonging to URM groups may perceive that combinations of their professional characteristics could indirectly reveal personal demographic characteristics as relatively few individuals in the profession may share the same combination of attributes. Thus, our findings suggest that perceived identifiability is not only driven by questions about personal identity, but also by professional and contextual characteristics that can distinguish individuals within a relatively small and interconnected workforce.

Sexuality (20.9%) and race/ethnicity (20.3%) were the most frequently selected underrepresented aspects of identity among GCs in our study. Additionally, participants who identified as a sexual minority reported a lower willingness to answer demographic questions regarding sexuality, while respondents identifying as racial/ethnic minorities reported lower willingness to answer race/ethnicity questions. While our study was not designed to determine the reasons for this difference, these findings suggest that concerns about disclosure may be heightened when individuals are asked to report demographic characteristics that correspond to their own marginalized identities. Previous research has shown that underrepresented racial and ethnic groups, including African American, Latine, Asian American, and Pacific Islander participants, are more likely than non‐Hispanic White participants to skip or decline demographic questions such as race, ethnicity, or gender (Dembosky et al. 2019; Srivastav et al. 2021). Similarly, respondents from racial and ethnic minority groups have been shown to be more likely to omit questions regarding sexual orientation in population‐based surveys (Kim and Fredriksen‐Goldsen 2013). Together, these findings are consistent with prior evidence that minoritized individuals often experience increased concerns about privacy, stigma, and potential discrimination when disclosing sensitive aspects of identity, particularly in professional or research settings. Intersectional identities, such as belonging to both a racial/ethnic and sexual minority, can further amplify these concerns, leading to reduced willingness to disclose or participate fully in research (Matalon et al. 2023).

Importantly, multiple participants in our study noted modifying their answers to demographic questions to increase perceived anonymity. While researchers often focus on unintentional sources of error, such as participant recall bias or misinterpretation of survey questions, our findings suggest that perceived identifiability may contribute to intentional response modifications. While the true frequency and impact of this behavior are unknown, such modifications can have implications for the reliability and validity of self‐reported demographic data and may obscure efforts to accurately categorize diversity within the profession and limit statistical analyses.

Beyond response modification, our findings suggest that perceptions of identifiability and the design of demographic questions may also influence broader attitudes toward research participation. Although all participants in our study expressed general willingness to engage in surveys and research, many questioned the perceived necessity and design of demographic questions and noted greater concern about identifiability when demographic characteristics were overly specific or combined. While our study did not directly examine reasons for nonparticipation in surveys, our data raises the possibility that perceived identifiability may influence decisions about whether to participate in research and surveys. The NSGC PSS has seen notable declines in response rates in recent years; the response rate to the PSS was estimated at 50% in 2020 and was down to 38% in 2024 (Campbell et al. 2024). While the specific drivers of reduced participation remain unclear, perceived identifiability may represent one potential contributing factor. Further research is needed to explore how survey design, perceived anonymity, and question burden influence research participation and the potential for nonresponse bias.

### 4.1 Limitations and Future Directions

While this study presents valuable insights, there are several notable limitations. The sample size was relatively small compared to the overall genetic counseling workforce, and most participants in our cohort reported that they regularly engage in surveys and research. Though the study was open to various members of the genetic counseling community, no Emeritus members of NSGC participated in the study. As a result, the concerns about identifiability reported in this study may not fully reflect the broader community, particularly those who less frequently participate in surveys and who practice or reside outside of the United States and Canada. Additionally, because participation was voluntary, there is an inherent risk of self‐selection bias. It is possible that GCs who had strong feelings or personal experiences related to demographic questions and identifiability in surveys were more likely to complete the survey, potentially influencing the results. We also let participants define for themselves what “underrepresented” meant in their identities. While this allowed personal expression, it may have introduced variability in how that term was interpreted. Future research could build on the current study to assess how attitudes toward identifiability and demographic questions may shift over time due to changes in professional norms, institutional practices, or other sociopolitical events.

## 5 Conclusion

Our study findings have practical implications for researchers conducting survey‐based studies within the genetic counseling profession and may also be relevant to researchers conducting interview‐based studies. Though collection of demographic information is still a critical part of research, researchers should carefully consider whether each demographic question asked is necessary and relevant to address the study question. Providing upfront rationale and transparency to clearly explain why specific demographic questions are being gathered and how anonymity will be preserved, particularly if small subsamples are expected, may help improve participant trust and reduce disclosure concern. Providing clear rationale for demographic questions, such as tailored explanations or infographics, can reduce item nonresponse among racially and ethnically diverse participants in self‐administered surveys (Lor et al. 2017). Researchers should also consider incorporating flexible response options for participants, including allowing multiple selections, having “prefer not to answer” options, and using open‐ended fields when appropriate. As reporting combinations of demographic characteristics or very small subgroup sizes may increase the risk of participant identification, researchers should thoughtfully assess how demographic data will be analyzed and reported and should consider strategies that may minimize direct identifiers, such as aggregating categories or suppressing small cell sizes when appropriate. These considerations may also be particularly relevant to research involving individuals living with genetic conditions, for whom combinations of demographic, clinical, and other personal characteristics may increase the potential for identifiability in both survey‐ and interview‐based research. As efforts to improve diversity and inclusion within the genetic counseling profession continue, thoughtful approaches to demographic data collection are essential to ensure that these efforts accurately reflect the identities of the communities they aim to represent while still protecting participant privacy.

## Author Contributions

A.R., J.C., and M.A. contributed substantially to the conceptualization of the study, development of the survey, collection of data, and review of the manuscript. A.R. and M.A. were also involved in data analysis and writing the original draft. All the authors gave final approval for publication of this version and agree to be accountable for all aspects of the work.

## Funding

The authors have nothing to report.

## Disclosure

The authors declare that generative AI was not used in the preparation of this manuscript.

## Ethics Statement

This study was approved by the Institutional Review Board at the University of Texas Health Science Center at Houston (HSC‐MS‐23‐0599).

## Conflicts of Interest

The authors declare no conflicts of interest.

## Data Availability

The data that support the findings of this study are available on request from the corresponding author. The data are not publicly available due to privacy or ethical restrictions.
